# Development and Validation of a Diagnostic Nomogram to Predict the Anthracycline-Induced Early Cardiotoxicity in Children with Hematological Tumors

**DOI:** 10.1007/s12012-022-09755-5

**Published:** 2022-06-16

**Authors:** Zhi-yuan Liu, Yun-gong Wang, Xiao-bi Huang, Xiao-hui Qi, Cui-ping Qian, Sheng Zhao

**Affiliations:** 1 Department of Cardiology, Anhui Provincial Children’s Hospital, Hefei, Anhui China; 2 The Fifth School of Clinical Medicine, Anhui Medical University, Hefei, Anhui China

**Keywords:** Cardiotoxicity, Anthracycline, Tumor, Nomogram, Hematologic, Children

## Abstract

This study aimed to establish and validate an effective nomogram to predict the risk of cardiotoxicity in children after each anthracycline treatment. According to the inclusion and exclusion criteria, the eligible children were randomly divided into the training cohort (75%) and the validation cohort (25%). Least absolute shrinkage and selection operator (LASSO) regression was used to select the predictors and a nomogram was developed. Then, concordance index (*C*-index), the area under the curve (AUC), Hosmer–Lemeshow (H–L) test, and decision curve analysis (DCA) were employed to evaluate the performance and clinical utility of nomogram. Internal validation was processed to inspect the stability of the model. A total of 796 eligible children were included in this study and divided into a training set (*n* = 597) and a validation set (*n* = 199). LASSO regression analysis revealed that cumulative anthracycline dose, ejection fractions, NT-proBNP, and diastolic dysfunction were effective predictors of cardiotoxicity. The nomogram was established based on these variables. The *C*-index and the AUC of the predicting nomogram were 0.818 in the training cohort and 0.773 in the validation cohort, suggesting that the nomogram had good discrimination. The calibration curve of the nomogram presented no significant deviation from the reference line, and the *P*-value of the H–L test was 0.283, implying a preferable degree of calibration. The threshold of DCA also reflects that the nomogram is clinically useful. A nomogram was developed to predict anthracycline chemotherapy-induced cardiotoxicity in children with hematological tumors. The nomogram has a good prediction effect and can provide a reference for clinicians’ diagnosis and treatment.

## Introduction

As an effective chemotherapeutic agent, anthracyclines are widely used in the treatment of various cancers, especially children’s hematological malignancies [1]. However, the cardiotoxicity caused by anthracyclines severely affects the quality of life of surviving cancer children and is significantly associated with a poor prognosis in these children [2]. Additionally, the clinical utility of anthracyclines is also significantly limited by their life-threatening cardiotoxic side effects [3]. Cardiovascular complications induced by anticancer therapy have become the main cause of morbidity and mortality in childhood cancer survivors [4].

To date, the specific mechanism of anthracycline-induced cardiotoxicity remains unclear. The mechanism of cardiotoxicity was mainly based on the hypothesis of cardiomyocyte oxidative stress, mitochondrial dysfunction, topoisomerase II β-dysfunction, and iron metabolism disorder [5–8]. Recent studies [9, 10] have demonstrated that early cardiac injury after anthracycline chemotherapy is transient and reversible. Early detection and prompt treatment may effectively lower long term of cardiovascular disease by preventing LV remodeling and progression to heart failure [11, 12]. However, it is more difficult to diagnose cardiotoxicity in clinical treatment owing to the unclear specific mechanism. Although the incidence of severe cardiotoxicity is dose dependent, even safely cumulative doses of anthracyclines may lead to permanent myocardial injury [13, 14]. Once clinical symptoms appear in children, even appropriate treatment cannot effectively prevent the progression of cardiotoxicity. Almost all children with heart failure need heart transplantation or die from cardiac complications [15]. Therefore, early identification and diagnosis of anthracycline-induced cardiotoxicity are essential, especially before permanent myocardial injury occurs.

Endomyocardial biopsies are still the gold standard for the diagnosis of myocardial injury induced by anthracycline. However, an invasive examination is not suitable for the large-scale detection of all children with anticancer therapy. At present, echocardiography is widely used in the clinical diagnosis of anthracycline cardiomyopathy [16]. Nevertheless, its sensitivity to screening at the early stage of heart injury is not high. Recently, some cardiac markers, such as N-terminal pro-B-type natriuretic peptide (NT-proBNP) and Troponin I (cTnT), are the focus of research and have the potential to diagnose cardiotoxicity before permanent heart injury. However, the diagnostic value of these cardiac markers in cardiotoxicity is obscure and even the conclusions of some studies are contradictory [17–19]. Considering the limited diagnostic ability of a single index, a multi-index summary model should be established for the early diagnosis of cardiotoxicity.

Nomogram is a prediction tool that can provide personalized disease-related risk estimation [20]. It constructs a multi-factor regression model, integrates the prediction indicators according to the regression coefficient of each influencing factor in the model, and then presents a visual prediction result. It has been broadly adopted to assist clinical professional decision-making, such as cancer prognosis, trauma treatment, and surgery [21–23]. To date, nomograms have not been applied for patients with anthracycline-induced cardiotoxicity. Our study aims to develop and validate a credible nomogram for predicting anthracycline-induced early myocardial injury during the treatment of hematological tumors in children.

## Methods

### Ethical Statement

The procedure followed in this study met the ethical standards formulated by the Ethics Committee of Anhui Children’s Hospital and was approved by the Committee. All patients received informed consent before treatment. Subjects or their agents gave informed verbal consent to participate in the study. The information provided by patients without disclosing personal privacy is protected.

### Patients and Study Design

Data from pediatric patients of the hematological tumor’s retrospective cohort of the Anhui Provincial Children’s Hospital (Hefei, China) treated with Anthracycline between January 1, 2014 and October 1, 2021 were retrospectively analyzed.

The inclusion criteria were described as follows: (1) patients who meet the diagnostic criteria of lymphohematopoietic system tumors revised by the World Health Organization in 2016 and confirmed by bone marrow puncture [24]; (2) patients aged 0–18 years old; (3) children with newly diagnosed blood tumor and standardized treatment with anthracyclines after diagnosis; and (4) no prior exposure to other cardiotoxic agents.

The exclusion criteria contained (1) children with congenital heart disease, such as ventricular septal defect, atrial septal defect, and valvular disease; (2) children with acquired heart disease, such as cardiomyopathy, myocarditis, arrhythmia, and pericardial disease; (3) the treatment was interrupted or the chemotherapy regimen was not strictly implemented; (4) inherited metabolic diseases, such as Down Syndrome; (5) dexrazoxane was used for protective treatment before the end of chemotherapy; and (6) patients with incomplete clinical data. Meanwhile, 3/4 children were randomly selected as modeling data, and the remaining 1/4 as validation data, so as to construct and verify the prediction model.

### Definition of Cardiotoxicity

The cardiac disorders in children were evaluated according to the standard for common terminology of adverse events (CTCAE) version 5.0 [25]. In our trial, cardiac toxicity was the primary endpoint and defined as follows: (i) a decrease in resting left ventricular ejection fraction (LVEF) of at least 20 points from baseline but remaining in the normal range, (ii) a decrease in LVEF of at least 10 points to below the lower limits of normal; or (iii) children experiencing clinical signs and/ or symptoms of CHF. All children included in the study had normal echocardiographic results before anthracycline treatment.

### Predictor Variables

Our nomogram was developed with the use of values of the predictors to predict cardiotoxicity after each chemotherapy cycle. Demographic information, cumulative dose of anthracyclines, imaging information, and related laboratory tests were collected from medical records that were scheduled after anthracycline chemotherapy. The body surface area (m^2^) is calculated by Dubois formula: (BSA = 0.007184 × *W*^0.425^ × *H*^0.725^; W: weight (kg); H: height (cm)) [26]. The types and cumulative doses of anthracyclines during chemotherapy were recorded. With daunorubicin as the actual cumulative dose, the various anthracyclines used in children were transformed according to the conversion coefficient of their equivalent dose of cardiotoxicity [27]. Finally, the cumulative dose of anthracyclines concerning body surface area was calculated based on the body surface area and cumulative dose of anthracyclines. Electrocardiogram (ECG) and echocardiography were detected after anthracycline treatment. The standard 12-lead body surface ECG (ECG-1550P, Japan Optoelectronics Corporation, Tokyo, Japan) was adopted for detection. Echocardiography (Philips iE33, Philips, Amsterdam, The Netherlands) was used to detect pericardial effusion. The end-diastolic and end-systolic left ventricular diameter, ejection fraction, and left ventricular shortening fraction were measured routinely. The results of the examination were judged by two doctors according to predetermined standards [28]. If the results were contradictory, the third researcher diagnosed after discussions.

cTnI/high-sensitivity cardiac troponin I and NT-proBNP were detected after each course of chemotherapy. Peripheral venous blood was collected in overnight fasting and quiet state and then measured by chemiluminescence immunoassay. The abnormal values of cTnI/high-sensitivity cardiac troponin I and NT-proBNP were > 0.06 ng/ml and > 125 pg/ml, respectively.

### Statistical Analysis

All data were analyzed by R (version 4.1.2). The packages involved are glmnet, rms, hmisc, and rmda. Since the criteria of echocardiography or ECG are different at different ages, the variables were classified before modeling. The cumulative dose (mg/m^2^) was divided into < 300, 300–550, and > 550 according to the research results [29–31]. The matching number of samples is more than 20 times the number of independent variables. In our study, some data of predictors are missing, which are processed by multiple imputations. The number of imputations is 64 times, and all prediction factors are included in the interpolation model. Continuous variables are expressed as mean ± standard deviation (SD) or median [interquartile range (IQR)]. *T* test or nonparametric rank-sum test were performed to compare the differences between the modeling group and validation group. Categorical variables are described by frequency; *χ*^2^ test or Fisher exact test was used to compare the differences between groups. The ‟glmnet” package of R software was utilized for least absolute shrinkage and selection operator (LASSO) regression analysis to screen variables. A nomogram model for predicting anthracycline cardiotoxicity in children with hematological malignancy was constructed according to the results of LASSO screening. The Harrell concordance index (*C*-index) analysis method was employed to evaluate the discrimination ability of the model and calculate the corresponding *C*-index. Then, the receiver operating characteristic (ROC) curve of the model in the modeling data set and validation data set is drawn to calculate the area under the curve (AUC). H–L (Hosmer–Lemeshow) test was conducted to evaluate the calibration degree of the prediction model. The bootstrap method was adopted (the number of sampling times was 1000 times) to verify the model and draw the calibration plots. Decision curve analysis (DCA) was performed in the modeling data set and validation data set to evaluate the clinical net benefit of the model [32]. *P* < 0.05 indicates statistically significant difference.

## Results

### Clinical Characteristics of Children with Anthracycline Chemotherapy

**Fig. 1:**
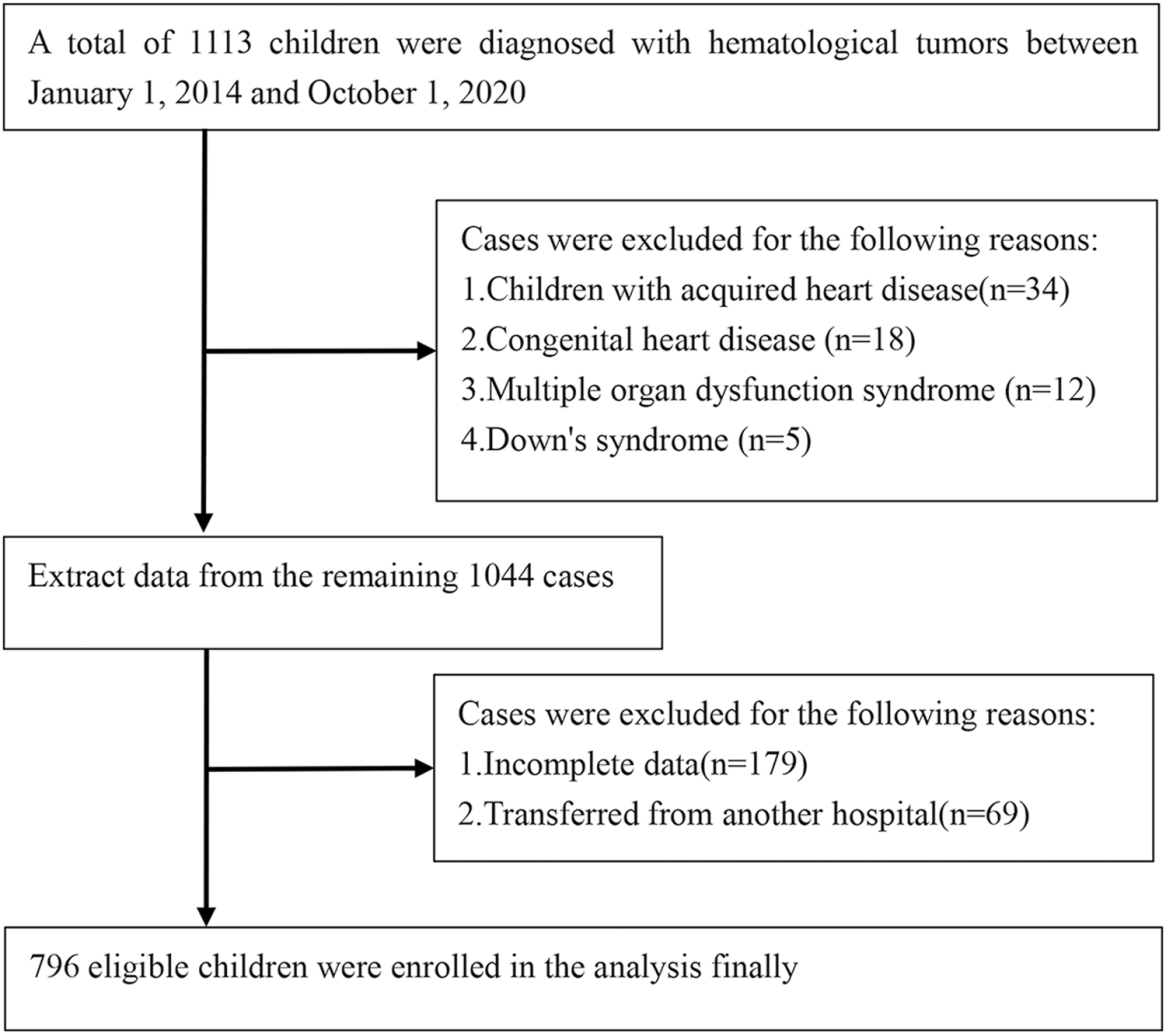
Flowchart of patient selection process based on the inclusion and exclusion criteria

According to the inclusion and exclusion criteria, we screened children with blood tumors who had used anthracycline chemotherapy between January 1, 2014 and October 1, 2021. Of the 1113 children diagnosed with blood tumors, 796 were finally included in our analysis (598 children in the training cohort and 199 children in the validation cohort). Among them, 9% of the children in the training cohort and 5% of the children in the validation cohort developed cardiotoxicity after anthracycline treatment. The specific screening process is illustrated in Fig. 1. Children in two cohorts had similar distributions of sex (*P* = 0.5564), age (*P* = 0.8437), type of malignancy (*P* = 0.7858), type of anthracycline (*P* = 0.4821), cumulative anthracycline dose (*P* = 0.9016), pericardial effusion (*P* = 0.6642), EF (*P* = 0.4252), cTnI (0.358), NT-proBNP (*P* = 0.7742), arrhythmia (*P* = 0.3218), and diastolic dysfunction (*P* = 0.8583). There were no significant differences between the two cohorts. The clinical characteristics of two cohort children are presented in Table 1.

**Table 1:** Baseline characteristics of pediatric patients with cancer

Patient characteristics	All patients *N* = 796	Training set *N* = 597	Validation set *N* = 199	*P* value
Age (years), *n* (%)				0.8437^a^
≤ 5	438 (55.03)	330 (55.28)	108 (54.27)	
6–10	268 (33.67)	198 (33.17)	70 (35.18)	
≥ 11	90 (11.31)	69 (11.56)	21 (10.55)	
Gender, *n* (%)				0.5564^a^
Male	488 (61.31)	362 (60.64)	126 (63.32)	
Female	308 (38.69)	235 (39.36)	73 (36.68)	
Type of malignancy, *n* (%)				0.7858^a^
ALL	601 (75.50)	448 (75.04)	153 (76.88)	
AML	152 (19.10)	115 (19.26)	37 (18.59)	
NHL	43 (5.40)	34 (5.70)	9 (4.52)	
Type of anthracycline, *n* (%)				0.4821^a^
Daunorubicin	253 (31.78)	193 (32.33)	60 (30.15)	
Doxorubicin	97 (12.19)	74 (12.40)	23 (11.56)	
Combine doxorubicin and daunorubicin	336 (42.21)	252 (42.21)	84 (42.21)	
Combine idarubicin and daunorubicin	79 (9.92)	53 (8.88)	26 (13.07)	
Combine pirarubicin and daunorubicin	31 (3.89)	25 (4.19)	6 (3.02)	
Cumulative anthracycline dose (mg/m^2^), *n* (%)				0.9016^a^
< 300	385 (48.37)	286 (47.91)	99 (49.75)	
300–550	342 (42.96)	259 (43.38)	83 (41.71)	
> 550	69 (8.67)	52 (8.71)	17 (8.54)	
Pericardial effusion, *n* (%)				0.6642^a^
No	749 (94.10)	560 (93.80)	189 (94.97)	
Yes	47 (5.90)	37 (6.20)	10 (5.03)	
EF (SD)	66 (62–69)	65 (62–69)	66 (63–71)	0.4252^b^
cTn I, *n* (%)				0.358^a^
Normal	745 (93.59)	562 (94.14)	183 (91.96)	
Abnormal	51 (6.41)	35 (5.86)	16 (8.04)	
NT-proBNP, *n* (%)				0.7742^a^
Normal	677 (85.05)	506 (84.76)	171 (85.93)	
Abnormal	119 (14.95)	91 (15.24)	28 (14.07)	
Arrhythmia, *n* (%)				0.3218^a^
Normal	779 (97.86)	582 (97.49)	197 (98.99)	
Abnormal	17 (2.14)	15 (2.51)	2 (1.01)	
Diastolic dysfunction, *n* (%)				0.8583^a^
Normal	687 (86.31)	514 (86.10)	173 (86.93)	
Abnormal	109 (13.69)	83 (13.90)	26 (13.07)	

*ALL* acute lymphoblastic leukemia, *AML* acute myeloid leukemia, *NHL* non-Hodgkin’s lymphoma, *EF* left ventricular ejection fraction, *SD* standard deviation, *cTn I* cardiac troponin I, *NT-proBNP* N-terminal pro-brain natriuretic peptide

^a^*P* value for Fisher exact test

^b^*P* value for Wilcoxon rank-sum test

### Predictors of Anthracycline-Induced Cardiotoxicity

**Fig. 2:**
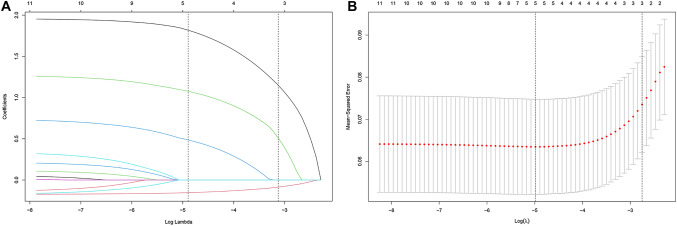
Feature selection using a least absolute shrinkage and selection operator (LASSO) regression model. **A** The LASSO coefficients of the 15 features. **B** Tuning parameter (*λ*) selection in the LASSO model used tenfold cross-validation using the minimal criteria. Dotted vertical lines were drawn at the *λ* minimum value

LASSO regression analysis was performed to explore the predictors of cardiotoxicity preliminarily. The results of regression analysis suggested four clinical variables with significant statistical significance in the training cohort. The predictors were cumulative anthracycline dose (coefficient 0.472), NT-proBNP (coefficient 1.799), EF (coefficient − 0.151), and diastolic dysfunction (coefficient 1.063). Dotted vertical lines were drawn at the *λ* minimum value (derived using the minimal criteria). The final *λ* value was 0.0075 and the log (*λ*) value was − 4.893. The coefficient profiles of all 11 features were included in the diagram (Fig. 2).

### Development of Predictive Nomograms for Cardiotoxicity

**Fig. 3:**
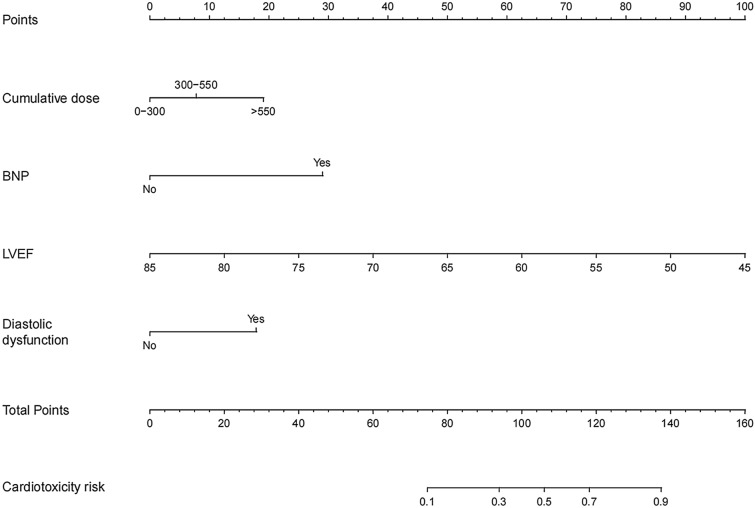
The nomogram for predicting the risk of anthracycline chemotherapy-induced cardiotoxicity in children with hematological tumors in the training cohort

The significant predictors screened by the LASSO regression analysis were integrated to build the nomogram for cardiotoxicity prediction. The predictive nomogram for anthracycline-induced cardiotoxicity is illustrated in Fig. 3. The first row (score) of the nomogram is the preliminary score for each variable, ranging from 0 to 100. Lines 2–5 represent a predictor of cardiotoxicity, respectively. Based on the scales in the axis of the nomogram, predicted scores of these variables can be calculated and then the scores of all variables are added to obtain the summation. A perpendicular line downward was drawn following the calculated total score, corresponding to the probability of cardiotoxicity in the last line (ranging from 0 to 100%). Therefore, each course of anthracycline chemotherapy reveals the children’s probability of myocardial damage within one year according to the nomogram.

### Verification of the Predictive Accuracy of the Nomogram

**Fig. 4:**
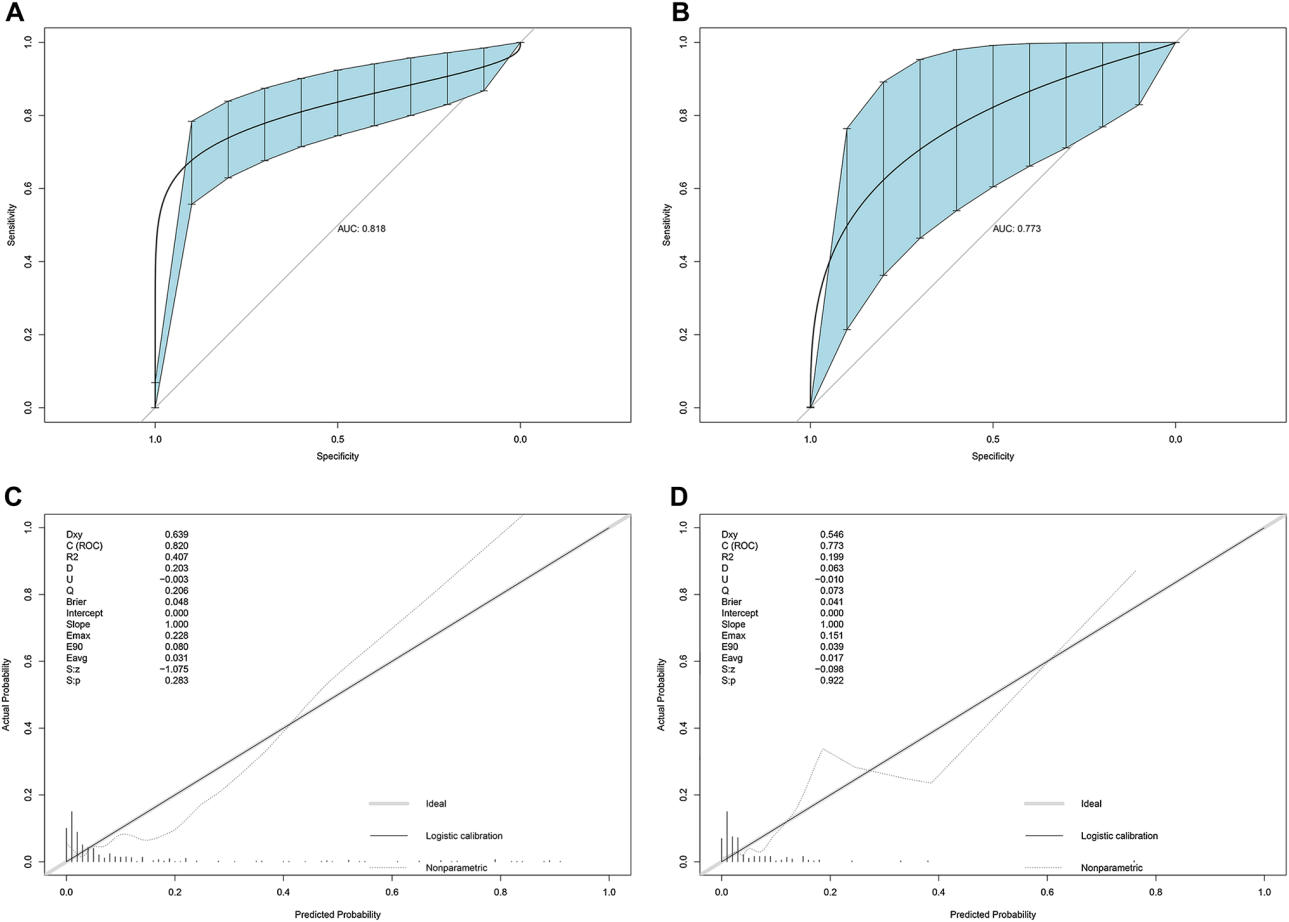
**A** The ROC curve of the nomogram for predicting the probability of anthracycline-induced cardiotoxicity in the training cohort. **B** The ROC curve of the nomogram for predicting the probability of anthracycline-induced cardiotoxicity in the validation cohort. **C** The calibration curve of nomograms for predicting anthracycline-induced cardiotoxicity in the training cohort. **D** The calibration curve of nomograms for predicting anthracycline-induced cardiotoxicity in the validation cohort

The *C*-index value and AUC were applied to evaluate the discrimination ability of our nomogram. Meanwhile, the *C*-index value and AUC were adjusted through 1000 bootstraps to ensure that the nomogram had good accuracy for predicting anthracycline-induced cardiotoxicity. The *C*-index of the predicting nomogram for anthracycline-induced cardiotoxicity was 0.818 in the training cohort and 0.773 in the validation cohort. As illustrated in Fig. 4, the AUC of the training cohort and the validation cohort was the same as their *C*-index values. The values of the *C*-index and AUC imply that the model performance between the two cohorts was relatively consistent. Thus, the nomogram has good discrimination ability. The cut-off value of the ROC curve was 0.238, suggesting that children would have a high risk of myocardial injury in future if the risk of cardiotoxicity is above 23.8%, according to the nomogram.

Moreover, the H–L test was conducted to evaluate the fitting degree of the model. As demonstrated by the calibration curve in Fig. 4, the predicted data of the training cohort and the validation cohort were in good agreement with the observed data within 1 year of anthracycline chemotherapy (training cohort *P* = 0.283; validation cohort *P* = 0.922). This reflects that the prediction probability of our model is close to the actual probability, and the nomogram has an acceptable calibration degree.

### Clinical Net Benefits of the Nomogram

**Fig. 5:**
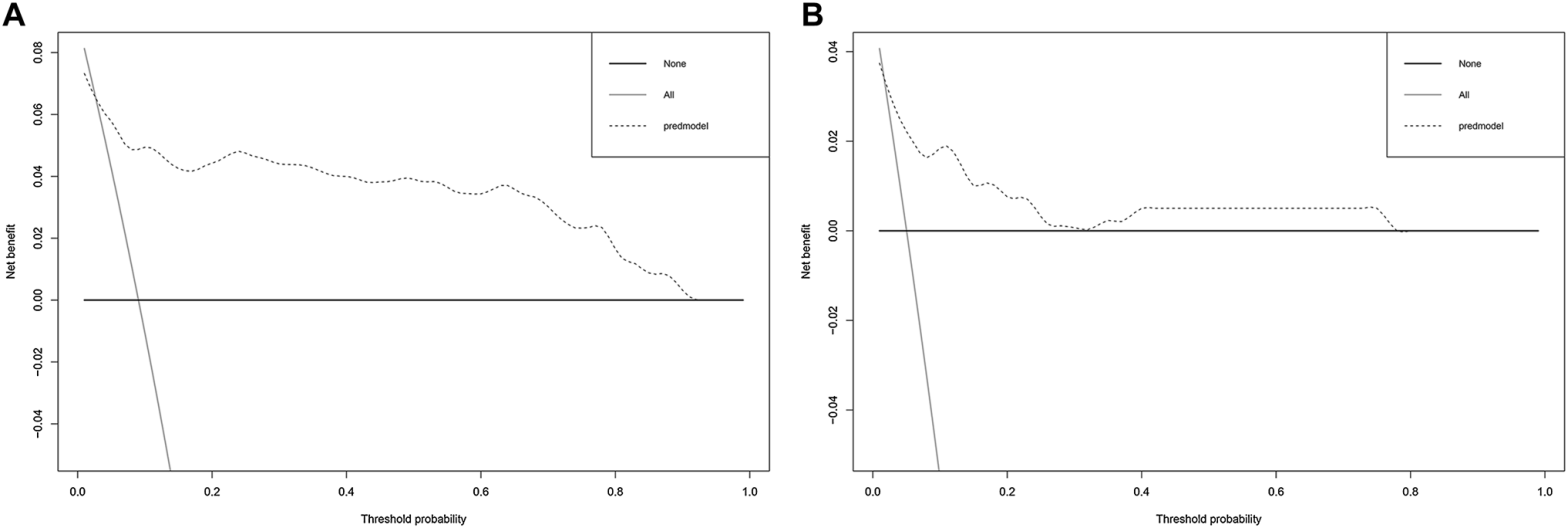
Decision curve analysis for the nomogram to predict anthracycline-induced cardiotoxicity. **A** Training cohort. **B** Validation cohort

The DCA was also conducted to evaluate the clinical usefulness of the nomogram for cardiotoxicity prediction. The DCA exhibited preferable net benefits of the nomogram along with the threshold probabilities in the training (Fig. 5A) and validation (Fig. 5B) cohorts. The decision curve illustrated that using this nomogram to predict the risk of cardiotoxicity is more beneficial than the scheme with or without intervention in all children if the threshold probability of children is > 1% and < 78% within one year of anthracycline chemotherapy. Within this range, the net benefit of the prediction nomogram is significantly higher than that of the two extreme treatment strategy, revealing that it has good clinical application significance in predicting anthracycline-induced cardiotoxicity.

## Discussion

In previous studies, there were several models for predicting cardiotoxicity in patients with anthracycline chemotherapy, although none of them were developed for hematological malignancies or children [33, 34]. At present, studies focus more on the assessment of risk factors and the exploration of predictors for cardiotoxicity. Nonetheless, the models set for predicting cardiotoxicity are different due to the different types of tumors and the difference in the study population. Although many studies have reported the related predictors of anthracycline chemotherapy-induced cardiotoxicity in children’s hematological malignancies, there is little research on the development and validation of a prediction model of cardiotoxicity based on these predictors. In this single-center, retrospective, and population-based study, a novel and practical nomogram was presented to predict the risk of cardiotoxicity in children. Compared with a single clinical predictor, this model can improve the prediction ability of early cardiac toxicity after anthracycline chemotherapy. Our study is the first nomogram to predict anthracycline chemotherapy-induced cardiotoxicity.

Nomogram is an intuitive visual tool for predicting disease and can convert a complex regression equation with clinical variables into a simple and visual graph [21]. Because of its readability and practicability, the nomogram is widely applied to the prediction of cancer prognosis and has attracted much attention in medical research and clinical decision-making in recent years [21–23]. In our study, 11 predictors were considered to narrow the bias of nomograms, such as demographic information, biomarkers, and imaging features. These variables were screened by LASSO regression analysis and then the final four predictors for cardiotoxicity were identified. Demographic variables mainly consist of age at cancer diagnosis, gender of children, type of tumor, and cumulative dose of anthracyclines related to body surface area. The age of children was divided into three grades: < 6 years old, 6–10 years old, and > 11 years old. Similarly, the dose–response relationship of anthracycline-induced cardiotoxicity is unclear in children. Therefore, the classified variable of cumulative dose of anthracyclines was used, instead of the continuous variable for regression analysis. According to the research results [28–30], the cumulative dose of anthracyclines of body surface area was divided into three grades: < 300 mg/m^2^, 300–550 mg/m^2^, and > 550 mg/m^2^. The regression analysis results revealed that sex, age at diagnosis, and type of tumor were not the predictors of cardiotoxicity, consistent with the study of Spewak et al. [35]. Subsequently, our results demonstrated that the cumulative dose of anthracyclines had a potential correlation with the occurrence of cardiotoxicity. It confirms observations from previous studies [9, 36]. Hence, children with a high cumulative dose of anthracycline chemotherapy may have a higher risk of cardiotoxicity than children with a low cumulative dose.

As a simple, repeatable, and risk-free diagnostic tool, cardiac biomarkers have great potential in early identification, evaluation, and monitoring of cardiotoxicity risk caused by anthracyclines. In the past 10 years, many cardiac biomarkers related to cardiotoxicity have been developed. However, the current research still stresses troponin I/HS-troponin and NT-proBNP, although they are full of disputes and contradictions in the conclusions of some studies [17–19]. Therefore, this study attempted to identify whether they have sufficient diagnostic ability in the early stage of cardiotoxicity. Notably, the reference values of these biomarkers may vary in childhood, and the standard values of children of different ages may be different. Additionally, the value of NT-proBNP decreased with age and increased in the second decade of girls [37, 38]. The NT-proBNP and cTnI were used as binary variables rather than continuous ones to reduce the possible bias of these confounding factors (such as age and race) on our research conclusions. LASSO regression analysis suggested that NT-proBNP played an important role in our nomogram, while the ability of troponin I as an early diagnostic cardiac biomarker was not enough. This result is consistent with the conclusion of a recent meta-analysis [19]. This meta-analysis included 27 related studies. The pooled data revealed that NT-proBNP is associated with cardiotoxicity in pediatric cancer patients receiving anthracycline therapy. However, the diagnostic benefit of troponin was unclear. In contrast, a study of 703 cancer patients demonstrated that elevated troponin in patients receiving cardiotoxic chemotherapy may be a sensitive indicator of early cardiotoxicity [39]. Other small studies have also confirmed the correlation between the increase of troponin and the decrease of LVEF [40, 41]. These contradictions may be correlated with the optimal timing of plasma marker assessment. NT-proBNP is mainly synthesized and secreted by left ventricular cardiomyocytes and regulated by left ventricular wall tension [42]. The severity of left ventricular dysfunction is positively correlated with its secretion. Diastolic dysfunction frequently occurs in children with cardiotoxicity [9], leading to more frequent abnormal secretion of NT-proBNP and more easily detected in peripheral blood. Troponin I is a specific index of myocardial injury. The peak time of cTnI after myocardial infarction is 10–24 h. The short detection time window usually induces false negatives in the detection of children’s peripheral blood. Owing to the exact timing of biomarker measurement and the variability of technology, their value in diagnosis has not been adequately affirmed. However, our nomogram fills these deficiencies to a great extent. Nomogram predicts cardiotoxicity by combining various variables, rather than relying on a single index or biomarker.

As a widely available, repeatable, and noninvasive tool for continuous evaluation of cardiac function, echocardiography is the most commonly used imaging technology to monitor cardiac function during and after chemotherapy. LVEF is a relatively insensitive tool in the diagnosis of the early stage of cardiotoxicity, since the decrease of LVEF does not occur until a critical number of myocardial injuries and the depletion of the cardiac compensation mechanism [43]. However, LVEF found almost all cardiotoxicity in the early stage in a recent large-scale study of breast cancer [9]. This study suggested that LVEF at the end of chemotherapy is an independent predictor of the development of cardiotoxicity. In patients with chemotherapy-induced cardiotoxicity, diastolic dysfunction may occur at an early stage [44]. Similarly, pericardial effusion, as a complication of anticycline, is related to cardiac dysfunction, which also implies pericardial disease and hemodynamic instability. This may be a sign of early adverse progression of cardiotoxicity [45]. Therefore, LASSO regression analysis was performed on LVEF, pericardial effusion, and diastolic dysfunction in this paper. The results demonstrated that LVEF and diastolic dysfunction were effective predictors of anthracycline-induced cardiotoxicity, with significant statistical significance.

Other predictors, such as arrhythmia, were not confirmed in the present research. Finally, the prediction model was established using binary logistic regression according to the results of LASSO regression analysis. The multivariate prediction equation involves the following four variables: cumulative anthracycline dose, LVEF, diastolic dysfunction, and NT-proBNP. Simultaneously, R software was adopted to convert this regression model into a nomogram. It is a tool for the diagnosis and prediction of anthracycline-induced cardiotoxicity. Besides, the ROC curve, H–L test, and DCA were conducted to evaluate the quality of the nomogram. The *C*-index value (0.818) and calibration curve implied that the nomogram performed well in the prediction of cardiotoxicity and had a preferable ability of discrimination and calibration. Additionally, internal verification was conducted, and the results demonstrated good consistency between the training cohort and the validation cohort. Thus, our results are stable and reliable. The DCA curves also reflected that clinical net benefits were produced by the nomogram in both of the training and validation sets. With the nomogram constructed in this study, clinicians can assess the cardiotoxicity risk of children with blood tumors. Clinicians can measure these readily available predictors after each chemotherapy and estimate the risk of cardiotoxicity through our nomogram. Clinicians can further check suspected high-risk children following the nomogram for early diagnosis and treatment. Moreover, they can selectively add cardioprotective agents such as dexrazoxane to prevent myocardial damage during chemotherapy. Generally, our nomogram might provide a simple and accurate prediction method for the diagnosis of anthracycline-induced cardiotoxicity.

Our study has several limitations. First, the number of factors leading to the study is limited because our study is retrospective. Some traditional variables, such as race and radiotherapy, and some novel predictors, such as GLS and tissue-type plasminogen activator, were not included in our study. Hence, a large prospective study is necessary. Second, this is a single-center study with data from a tertiary hospital. The number of cases is limited and not as representative as multicenter studies, although unified diagnostic criteria are beneficial to our study and analysis. Third, our study lacks external validation. Although the model exhibits good predictive performance in our study, it may not perform well in external validation in other child cohorts. Whether the model can be extended to primary hospitals should be further verified with more external data.

## Conclusion

Our study validated some potential predictors of cardiotoxicity after chemotherapy in children with hematological tumors and established a concise and convenient nomogram based on these factors. Our nomogram, as the first tool to predict cardiotoxicity after anthracycline chemotherapy, has good discrimination and calibration ability, as demonstrated by good *C*-index and calibration plots. Concurrently, DCA suggests that the model has acceptable clinical application prospects and can provide effective decision-making for clinicians after each anthracycline treatment. In future, some prospective, multicenter, and externally validated studies should be conducted to improve the model and expand its applicable population.

## Data Availability

The data sets generated during and/or analyzed during the current study are available from the corresponding author on reasonable request.

## References

[1] Singal PK, Iliskovic N (1998). Doxorubicin-induced cardiomyopathy. New England Journal of Medicine.

[2] Grenier MA, Lipshultz SE (1998). Epidemiology of anthracycline cardiotoxicity in children and adults. Seminars in Oncology.

[3] Pai VB, Nahata MC (2000). Cardiotoxicity of chemotherapeutic agents: Incidence, treatment and prevention. Drug Safety.

[4] Kremer LC, Caron HN (2004). Anthracycline cardiotoxicity in children. New England Journal of Medicine.

[5] Horenstein MS, Vander Heide RS, L'Ecuyer TJ (2000). Molecular basis of anthracycline-induced cardiotoxicity and its prevention. Molecular Genetics and Metabolism.

[6] Link G, Tirosh R, Pinson A, Hershko C (1996). Role of iron in the potentiation of anthracycline cardiotoxicity: Identification of heart cell mitochondria as a major site of iron–anthracycline interaction. Journal of Laboratory and Clinical Medicine.

[7] Ichikawa Y, Ghanefar M, Bayeva M, Wu R, Khechaduri A, Naga Prasad SV (2014). Cardiotoxicity of doxorubicin is mediated through mitochondrial iron accumulation. Journal of Clinical Investigation.

[8] Zhang S, Liu X, Bawa-Khalfe T, Lu LS, Lyu YL, Liu LF (2012). Identification of the molecular basis of doxorubicin-induced cardiotoxicity. Nature Medicine.

[9] Cardinale D, Colombo A, Bacchiani G, Tedeschi I, Meroni CA, Veglia F (2015). Early detection of anthracycline cardiotoxicity and improvement with heart failure therapy. Circulation.

[10] Kobayashi S, Volden P, Timm D, Mao K, Xu X, Liang Q (2010). Transcription factor GATA4 inhibits doxorubicin-induced autophagy and cardiomyocyte death. Journal of Biological Chemistry.

[11] Plana JC, Galderisi M, Barac A, Ewer MS, Ky B, Scherrer-Crosbie M (2014). Expert consensus for multimodality imaging evaluation of adult patients during and after cancer therapy: A Report from the American Society of Echocardiography and the European Association of Cardiovascular Imaging. European Heart Journal: Cardiovascular Imaging.

[12] Mulrooney DA, Yeazel MW, Kawashima T, Mertens AC, Mitby P, Stovall M (2009). Cardiac outcomes in a cohort of adult survivors of childhood and adolescent cancer: Retrospective analysis of the Childhood Cancer Survivor Study cohort. BMJ.

[13] Lipshultz SE, Sanders SP, Goorin AM, Krischer JP, Sallan SE, Colan SD (1994). Monitoring for anthracycline cardiotoxicity. Pediatrics.

[14] Lipshultz SE, Rifai N, Dalton VM, Levy DE, Silverman LB, Lipsitz SR (2004). The effect of dexrazoxane on myocardial injury in doxorubicin-treated children with acute lymphoblastic leukemia. New England Journal of Medicine.

[15] Alvarez JA, Scully RE, Miller TL, Armstrong FD, Constine LS, Friedman DL (2007). Long-term effects of treatments for childhood cancers. Current Opinion in Pediatrics.

[16] van Dalen EC, van den Brug M, Caron HN, Kremer LC (2006). Anthracycline-induced cardiotoxicity: Comparison of recommendations for monitoring cardiac function during therapy in paediatric oncology trials. European Journal of Cancer.

[17] Herman EH, Zhang J, Lipshultz SE, Rifai N, Chadwick D, Takeda K (1999). Correlation between serum levels of cardiac troponin-T and the severity of the chronic cardiomyopathy induced by doxorubicin. Journal of Clinical Oncology.

[18] Dodos F, Halbsguth T, Erdmann E, Hoppe UC (2008). Usefulness of myocardial performance index and biochemical markers for early detection of anthracycline-induced cardiotoxicity in adults. Clinical Research in Cardiology.

[19] Michel L, Mincu RI, Mrotzek SM, Korste S, Neudorf U, Rassaf T (2020). Cardiac biomarkers for the detection of cardiotoxicity in childhood cancer—A meta-analysis. ESC Heart Failure.

[20] Kattan MW (2002). Nomograms. Introduction. Seminars in Urologic Oncology.

[21] Balachandran VP, Gonen M, Smith JJ, DeMatteo RP (2015). Nomograms in oncology: More than meets the eye. Lancet Oncology.

[22] Langsetmo L, Nguyen TV, Nguyen ND, Kovacs CS, Prior JC, Center JR (2011). Independent external validation of nomograms for predicting risk of low-trauma fracture and hip fracture. CMAJ.

[23] Fong Y (2019). Textbook outcome nomograms as multivariate clinical tools for building cancer treatment pathways and prognosticating outcomes. JAMA Surgery.

[24] Quintanilla-Martinez L (2017). The 2016 updated WHO classification of lymphoid neoplasias. Hematological Oncology.

[25] U.S. Department of Health and Human Services, National Institutes of Health, National Cancer Institute. (2017). *Common terminology criteria for adverse events (CTCAE) v5.0*. U.S. Department of Health and Human Services, National Institutes of Health, National Cancer Institute. https://ctep.cancer.gov

[26] Du Bois, D., & Du Bois, E. F. (1916). A formula to estimate the approximate surface area if height and weight be known. *Nutrition 1989, 5*(5), 303–311; Discussion 312–213. 2520314

[27] Keefe DL (2001). Anthracycline-induced cardiomyopathy. Seminars in Oncology.

[28] Lopez, L., Colan, S. D., Frommelt, P. C., Ensing, G. J., Kendall, K., Younoszai, A. K., et al. (2010). Recommendations for quantification methods during the performance of a pediatric echocardiogram: A Report from the Pediatric Measurements Writing Group of the American Society of Echocardiography Pediatric and Congenital Heart Disease Council. *Journal of American Society of Echocardiography, 23*(5), 465–495; quiz 576–577. 10.1016/j.echo.2010.03.019 20451803

[29] Krischer JP, Epstein S, Cuthbertson DD, Goorin AM, Epstein ML, Lipshultz SE (1997). Clinical cardiotoxicity following anthracycline treatment for childhood cancer: The Pediatric Oncology Group experience. Journal of Clinical Oncology.

[30] Nysom K, Holm K, Lipsitz SR, Mone SM, Colan SD, Orav EJ (1998). Relationship between cumulative anthracycline dose and late cardiotoxicity in childhood acute lymphoblastic leukemia. Journal of Clinical Oncology.

[31] Rathe M, Carlsen NL, Oxhøj H, Nielsen G (2010). Long-term cardiac follow-up of children treated with anthracycline doses of 300 mg/m^2^ or less for acute lymphoblastic leukemia. Pediatric Blood Cancer.

[32] Vickers AJ, Elkin EB (2006). Decision curve analysis: A novel method for evaluating prediction models. Medical Decision Making.

[33] Upshaw JN, Ruthazer R, Miller KD, Parsons SK, Erban JK, O'Neill AM (2019). Personalized decision making in early stage breast cancer: Applying clinical prediction models for anthracycline cardiotoxicity and breast cancer mortality demonstrates substantial heterogeneity of benefit–harm trade-off. Clinical Breast Cancer.

[34] Dranitsaris G, Rayson D, Vincent M, Chang J, Gelmon K, Sandor D (2008). The development of a predictive model to estimate cardiotoxic risk for patients with metastatic breast cancer receiving anthracyclines. Breast Cancer Research Treatment.

[35] Spewak MB, Williamson RS, Mertens AC, Border WL, Meacham LR, Wasilewski-Masker KJ (2017). Yield of screening echocardiograms during pediatric follow-up in survivors treated with anthracyclines and cardiotoxic radiation. Pediatric Blood Cancer.

[36] Lotrionte M, Biondi-Zoccai G, Abbate A, Lanzetta G, D'Ascenzo F, Malavasi V (2013). Review and meta-analysis of incidence and clinical predictors of anthracycline cardiotoxicity. American Journal of Cardiology.

[37] Nir A, Lindinger A, Rauh M, Bar-Oz B, Laer S, Schwachtgen L (2009). NT-pro-B-type natriuretic peptide in infants and children: Reference values based on combined data from four studies. Pediatric Cardiology.

[38] Koch AM, Rauh M, Zink S, Singer H (2006). Decreasing ratio of plasma N-terminal pro-B-type natriuretic peptide and B-type natriuretic peptide according to age. Acta Paediatrica.

[39] Cardinale D, Sandri MT, Colombo A, Colombo N, Boeri M, Lamantia G (2004). Prognostic value of troponin I in cardiac risk stratification of cancer patients undergoing high-dose chemotherapy. Circulation.

[40] Specchia G, Buquicchio C, Pansini N, Di Serio F, Liso V, Pastore D (2005). Monitoring of cardiac function on the basis of serum troponin I levels in patients with acute leukemia treated with anthracyclines. Journal of Laboratory and Clinical Medicine.

[41] Auner HW, Tinchon C, Linkesch W, Tiran A, Quehenberger F, Link H (2003). Prolonged monitoring of troponin T for the detection of anthracycline cardiotoxicity in adults with hematological malignancies. Annals of Hematology.

[42] Hall C (2004). Essential biochemistry and physiology of (NT-pro)BNP. European Journal of Heart Failure.

[43] Ewer MS, Lenihan DJ (2008). Left ventricular ejection fraction and cardiotoxicity: Is our ear really to the ground?. Journal of Clinical Oncology.

[44] Nagueh SF, Appleton CP, Gillebert TC, Marino PN, Oh JK, Smiseth OA, Waggoner AD, Flachskampf FA, Pellikka PA, Evangelisa A (2009). Recommendations for the evaluation of left ventricular diastolic function by echocardiography. European Journal of Echocardiography.

[45] Galderisi M, Marra F, Esposito R, Lomoriello VS, Pardo M, de Divitiis O (2007). Cancer therapy and cardiotoxicity: The need of serial Doppler echocardiography. Cardiovascular Ultrasound.

